# Bioprospecting for Exopolysaccharides from Deep-Sea Hydrothermal Vent Bacteria: Relationship between Bacterial Diversity and Chemical Diversity

**DOI:** 10.3390/microorganisms5030063

**Published:** 2017-09-20

**Authors:** Christine Delbarre-Ladrat, Marcia Leyva Salas, Corinne Sinquin, Agata Zykwinska, Sylvia Colliec-Jouault

**Affiliations:** 1 Ifremer, Laboratoire Ecosystèmes Microbiens et Molécules Marines pour les Biotechnologies, Rue de l’Ile d’Yeu, BP 21105, 44311 Nantes, France; corinne.sinquin@ifremer.fr (C.S.); Agata.Zykwinska@ifremer.fr (A.Z.); sylvia.colliec.jouault@ifremer.fr (S.C.-J.); 2 Givaudan France SAS, 51110 Pomacle, France; marcialeyvas@gmail.com

**Keywords:** exopolysaccharides, GAG, marine bacteria, glycopolymers, sulfate, production

## Abstract

Many bacteria biosynthesize structurally diverse exopolysaccharides (EPS) and excrete them into their surrounding environment. The EPS functional features have found many applications in industries such as cosmetics and pharmaceutics. In particular, some EPS produced by marine bacteria are composed of uronic acids, neutral sugars, and *N*-acetylhexosamines, and may also bear some functional sulfate groups. This suggests that they can share common structural features with glycosaminoglycans (GAG) like the two EPS (HE800 and GY785) originating from the deep sea. In an attempt to discover new EPS that may be promising candidates as GAG-mimetics, fifty-one marine bacterial strains originating from deep-sea hydrothermal vents were screened. The analysis of the EPS chemical structure in relation to bacterial species showed that *Vibrio*, *Alteromonas*, and *Pseudoalteromonas* strains were the main producers. Moreover, they produced EPS with distinct structural features, which might be useful for targeting marine bacteria that could possibly produce structurally GAG-mimetic EPS.

## 1. Introduction

Glycosaminoglycans (GAGs) are structurally diverse animal glycopolymers with key roles in cell physiology and in some pathologies. These polysaccharides are polyanionic macromolecules composed of alternating uronic acids and hexosamines or neutral sugars [1,2]. Upon biosynthesis, GAG backbones undergo a series of specific enzymatic reactions leading to a large variety of *N*- and *O*-sulfations and epimerization patterns; these molecules are also heterogeneous with respect to size [3].

Hyaluronan (HA) is a non-sulfated and high molecular weight polymer. It is used in osteoarthritis treatment, in ophthalmology, and in wound healing as well as in the cosmetic industry due to its visco-elastic properties and biological properties on the cartilage and skin [1,4]. In contrast to HA, other GAGs (heparan, dermatan, chondroitin, and keratan sulfate) are highly sulfated; this remains crucial in some biological activities such as in anti-coagulation, cell-proliferation, and inflammation, as well as in fighting viruses, prions, Alzheimer’s and Parkinson’s diseases [1]. Heparin is a leading drug against many diseases and has been used medically for over 70 years [5]. However, finding alternative molecules is necessary as heparin may exhibit severe side effects, often due to contamination during its extraction [5,6,7]. Although the pentasaccharide unit, which bears both anti-coagulant and anti-thrombotic activities, was obtained through chemical synthesis and is now commercially available, it may still induce some undesirable bleeding reactions [8,9].

Polysaccharides are ubiquitous, not only in animal cells but in all other living cells. They can be produced by microorganisms, particularly bacteria. In the case of marine bacteria, excreted polysaccharides, in particular exopolysaccharides (EPS), constitute molecules important for cell survival [10]. They mediate interaction with abiotic surfaces as well as with bacterial counterparts and other organisms [11]. EPS may also protect bacterial cells from harsh conditions [12] and trap limited nutrients to provide them to the cells [13]. Marine EPS are characterized by a large diversity of chemical compositions and structures, which can be exploited to gain compounds with innovative properties, in particular GAG-mimetic ones [14].

Bacterial cultures offer many advantages to obtain large amounts of putative therapeutically high value compounds. Cultivation in bioreactors allows for the renewable and controlled production of polysaccharides as their extraction is easier than animal or plant counterparts, and the obtained products are not subjected to physiological or seasonal fluctuations. Moreover, the risk of contamination by viruses, allergenic animal proteins, or toxic compounds is lower in bacterial extracts than in those from animals. Indeed, when extracted from animal sources, polysaccharides are typically isolated from connective tissue e.g., porcine mucosa for heparin, bovine cartilage for chondroitin sulfate, and rooster combs for HA. Therefore, bacteria are particularly attractive sources to develop innovative GAG-mimetic compounds for human health and well-being.

*Escherichia coli* K4 and K5 strains have been shown to produce polysaccharides (named K4 and K5, respectively) that have the same structure as the non-sulfated precursors in the chondroitin sulfate and heparan sulfate biosynthesis, respectively [15,16]. Thus, they have to be sulfated to enhance their GAG-mimetic activities [17] as they do not bear any sulfate groups. On the other hand, some marine bacteria have been shown to produce naturally sulfated polysaccharides [18,19,20,21], which can advantageously mimic GAG from animal origins [22]. Chemical or enzymatic modifications of these produced molecules can also be conducted through a semi-synthetic process to enhance the GAG-like activities [23,24]. Recently, a depolymerized and oversulfated derivative of the EPS produced by *Alteromonas infernus* was shown to be active in bone biology and have an anti-metastatic effect on osteosarcoma [25,26]. On the other hand, the native HE800 EPS produced by *Vibrio diabolicus* has demonstrated its efficiency in bone and skin regeneration [27,28].

In this study, we investigated the ability of bacteria from deep-sea hydrothermal vents to produce EPS. The chemical composition of secreted EPS was assessed and discussed in relation to the phylogenetic position of the producing strain.

## 2. Experimental Section

### 2.1. Bacterial Strains

Strains (*n* = 51) were isolated from deep-sea hydrothermal vents samples collected during the Ifremer oceanographic cruises Starmer (ST), Hero (HE), Guaynaut (GY), Biolau (BI), Hydronaut (HY), Microsmoke (MS), and Marvel (MV) (Table S1). Strain names started with the two letters of the sampling cruise. *Vibrio diabolicus* [29] and *Alteromonas infernus* [30] were also added to this study. Bacterial strains were stored at −80 °C in 20% (*v*/*v*) glycerol.

### 2.2. Culture Media

The mesophilic and aerobic bacteria were grown on Zobell (aquarium salts 33.3 g·L^−1^, yeast extract (Bacto) 1.0 g·L^−1^, Tryptone N1 (OrganoTechnie) 4.0 g·L^−1^). For EPS production, a modified Zobell medium, named ZHPUF medium, was used. In this medium, the Bacto yeast extract was replaced by an ultrafiltered yeast extract (Ref 19,712, OrganoTechnie, La Courneuve, France) to remove carbohydrates that may influence EPS production. The ZHPUF medium was buffered by a mixture of 100 mM HEPES and PIPES (Sigma Aldrich, St. Louis, MO, USA) and pH was adjusted to 7.5. Glucose, saccharose, or mannitol was sterilized separately and added at a final concentration of 30 g·L^−1^. Incubation temperature was set to 25 °C or 37 °C (Table S1). Liquid cultures were agitated at 150 rpm.

### 2.3. EPS Production

Preliminary screening of EPS production was performed in 1.2 mL tubes containing 1 mL of culture. Samples were incubated at 30 °C under 150 rpm agitation during 48 h. Growth was confirmed by absorbance at 600 nm. Cultures were centrifuged at 15,000 rpm, 30 min, 4 °C (Allegra™ 25R, Beckman Coulter, Brea, CA, USA). A 500 µL sample of the supernatant was withdrawn and stored at −20 °C before analysis on agarose gel stained with Stains all (Sigma Aldrich, St. Louis, MO, USA), as previously described in Ref. [31].

EPS extraction and purification was carried out on 100 mL of culture in a 500 mL Erlenmeyer incubated at 30 °C, 150 rpm during 48 h. After incubation, each culture was centrifuged at 8000× *g*, 4 °C during 30 min (Avanti J-E Beckman Coulter, Villepinte, France). Supernatant was filtered under vacuum on 2.6 µm and 0.7 µm glass microfiber membranes (Whatman, Maidstone, UK), purified by tangential ultrafiltration with a membrane of 100 kDa cut-off (Millipore, VWR, Fontenay-sous-Bois, France) and freeze dried. Production yield was estimated by the weight of the recovered powder.

### 2.4. Chemical Analysis

EPS solutions were prepared at 2 mg·mL^−1^ in water under very soft overnight agitation. The protein, sugar, and sulfur contents were determined by the corresponding method as follows. Co-extracted proteins were analyzed using the bicinchoninic acid method (BCA-Kit, Sigma). Neutral sugars were assessed by the method of Dubois et al. described in Reference [32] using glucose as the standard. Uronic acids were determined using the method proposed by Blumenkrantz and Asboe-Hansen [33], modified by Filisetti-Cozzi and Carpita [34]. The neutral sugar amount was corrected by the method of Montreuil and Spik [35] to consider the interfering uronic acid sugars. Sulfur content, indicating the presence of sulfate groups, was semi-quantitatively estimated by Azure A (Sigma) as follows. The EPS solution (50 µL) was mixed with 1 mL Azure A at 10 µg/mL in water. Absorbance at 535 nm gave the sulfur content (% *w*/*w*) based on an in-lab EPS reference whose sulfur content was determined by elemental analysis (Institut de Chimie des Substances Naturelles, ICSN-CNRS, Gif-sur-Yvette, France). Monomer compositions were determined by gas chromatography (GC) as per Karmeling et al. [36], modified by Montreuil et al. [37].

### 2.5. Molecular Weight Determination

The weight-average molecular weight was determined by high-performance size-exclusion chromatography (HPSEC) with multiangle light scattering (MALS, Dawn Heleos-II, Wyatt Technology Sc, Toulouse, France) and differential refractive index (Optilab, Wyatt Technology Sc, Toulouse, France) detectors. The HPSEC system was composed of an HPLC system (Prominence Shimadzu, Marne la Vallée, France) a PL aquagel-OH mixed, 8 µm (Agilent Technologies France S.A.S, Massy, France) guard column (50 × 7.5 mm), and a PL aquagel-OH mixed (Agilent Technologies France S.A.S, Massy, France) separation column (300 × 7.5 mm, operating range: 10^2^–10^7^ g·mol^−1^). EPS samples were filtered on a 0.45 µm cellulose acetate 4 mm syringe filter prior to injection (100 μL). Elution was performed at 1 mL·min^−1^ with 0.1 M ammonium acetate containing 0.03% NaN_3_, filtered on 0.1 µm membrane (Durapore Membrane, PVDF, Hydrophilic type VVLP, Millipore, Billerica, MA, USA). Data were computed with Astra 6.1 software (Wyatt Technology Sc, Toulouse, France) for absolute molar mass determinations. The molecular weight was calculated using a specific refractive index increment (d*n*/d*c*) value of 0.145 mL·g^−1^.

### 2.6. Phylogenetic Analysis

16S rRNA genes were amplified by PCR (Polymerase Chain Reaction) on a whole cell extract prepared as follows. A 500 µL culture was centrifuged at 10,000 rpm, 4 °C. The bacterial cell pellet was washed with PBS (Phosphate Buffer Saline) and subsequently suspended in 40 µL of sterile water. Samples were stored at −20 °C until analysis.

PCR were carried out with GoTaq ColorlessMasterMix 2× (Promega) on ten-time diluted samples with 55 °C hybridization and 1.5 min elongation using appropriate primers (8F (5′-AGAGTTTGATCATGGCTCAG-3′) and 1489R (5′-GTTACCTTGTTACGACTTCAC-3′)). All of the PCR products were run on a 1% agarose gel electrophoresis with Sybr safe (Invitrogen, Fisher Scientific, Illkirch, France) and purified or extracted from gel with the GeneJET Gel Extraction and DNA Cleanup Micro Kit (Thermofisher, Waltham, MA, USA) before sequencing (GATC Biotech, Konstanz, Germany). A consensus sequence was built using forward and reverse sequences with Bioedit software [38]. Strain identification was performed by nucleotide Blast (megablast program) on the NCBI nr/nt Nucleotide Collection [39].

A phylogenetic tree was established using sequenced 16S rRNA genes. Reference strain 16S rRNA sequences were retrieved from NCBI (Table S3). Sequences were aligned with ClustalW and phylogenetic analyses were conducted with MEGA6 software [40]. The evolutionary history was inferred using the Neighbor-Joining method [41], and the optimal tree shown. The percentage of replicate trees where the associated taxa clustered together in the bootstrap test (1000 replicates) were shown next to the branches [42]. The tree was drawn to scale, with branch lengths in the same units as those of the evolutionary distances used to infer the phylogenetic tree. The evolutionary distances were computed using the Jukes–Cantor method [43] and were in the units of the number of base substitutions per site. The analysis involved 40 nucleotide sequences. All ambiguous positions were removed for each sequence pair. *E. coli* was chosen as the outgroup.

## 3. Results and Discussion

### 3.1. Production of Exopolysaccharide

In this work, 51 strains isolated from deep-sea hydrothermal vents were analyzed for their polysaccharide production ability (Table S1). They were selected after a preliminary screening on a solid medium for mucoid colony formation indicating the putative ability of the bacterial strain to biosynthesize EPS (data not shown).

**Figure 1:**
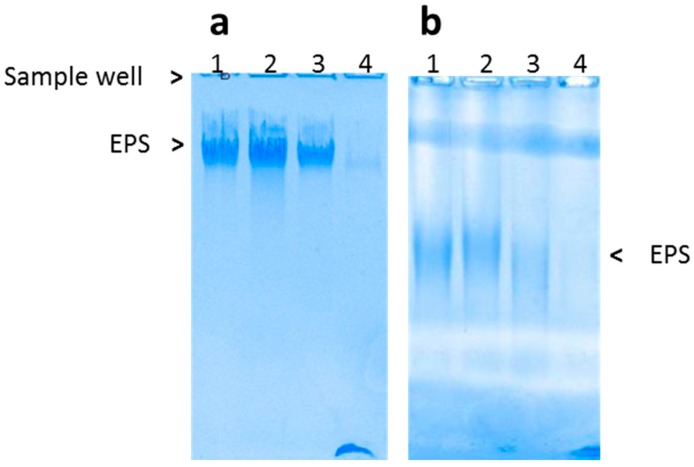
Examples of EPS production visualized by agarose gel electrophoresis (**a**) MA896B; (**b**) HE799. Growth medium: (1) glucose, (2) saccharose, (3) mannitol, and (4) ZPHUF alone.

Bacteria were grown on liquid ZPHUF medium supplemented with glucose, saccharose, or mannitol (30 g·L^−1^). Both *Alteromonas infernus* and *Vibrio diabolicus,* producing GY785 and HE800 EPS, respectively, were used as laboratory references [44,45]. EPS released outside the cell were visualized by agarose gel electrophoresis (Figure 1). EPS were produced in larger amounts when glucose or saccharose was used. A basal production was also observed on the ZPHUF medium. The electrophoretic migration depends not only on molecular weight, but also on the charge of the EPS molecule that appeared relatively homogeneous in the four different media, suggesting that there were no major variations in molecular weight and global charge regardless of the carbon source used. As observed in Figure 1, the MA896B and HE799 strains produced two different EPS whose electrophoretic migration clearly differed.

EPS molecules revealed by stains with all coloration adapted for acidic polysaccharides were found in 42 strains including *A. infernus* and *V. diabolicus*, i.e., 79% of the screened strains. There was no EPS production in 11 strains; moreover, 11 of the 42 positive strains produced EPS in very low amounts and four of them produced low molecular weight (LMW) EPS (Table S1). It could not be excluded that growth and production optimization could allow a better production yield of high-molecular weight (HMW) EPS from all the strains used in this study, but this possibility was not further investigated in this screening step.

The 27 strains producing high amounts of EPS were selected to be grown in a 100 mL culture (Table S1) including *A. infernus* and *V. diabolicus*. From this culture volume, we expected a sufficient amount of EPS to conduct chemical analyses. Moreover, through better agitation, we expected a better oxygen transfer rate, which was important for the EPS production yield [46,47]. An EPS was detected in the ZPHUF supplemented with glucose for all the selected strains with a good yield; consequently, glucose was chosen as the growth substrate for further studies. Indeed, glucose is a widespread substrate for fermentation bioprocesses in industry and would allow an easier scale up of the production process.

### 3.2. Chemical Characteristics of the EPS

Recovery yield and chemical composition (monosaccharide, protein, and sulfur contents) of the EPS produced by the different strains were determined (Table S2). Yield varied from 0.1 g·L^−1^ for MS919 and reached the highest yield of 0.58 g·L^−1^ for HY766. In comparison, in 100 mL culture, EPS produced by *V. diabolicus* and *A. infernus* were recovered with similar yields of 0.35 g·L^−1^ for HE800 EPS and 0.55 g·L^−1^ for GY785 EPS. This is consistent with the production yield usually encountered for marine EPS, which is around 1 g·L^−1^ [48]. However, the recovery yields of EPS from these reference strains obtained in bioreactors were considerably higher: 2.5 g·L^−1^ for HE800 EPS [29] and 5.5 g·L^−1^ for GY785 EPS [30]. Indeed, growth parameters were well-controlled in bioreactors. In particular, pH decreased when glucose was consumed, usually reaching 5 and resulted with lower growth in flasks. Although culture medium was buffered in our study, pH slightly decreased to 6.

**Figure 2:**
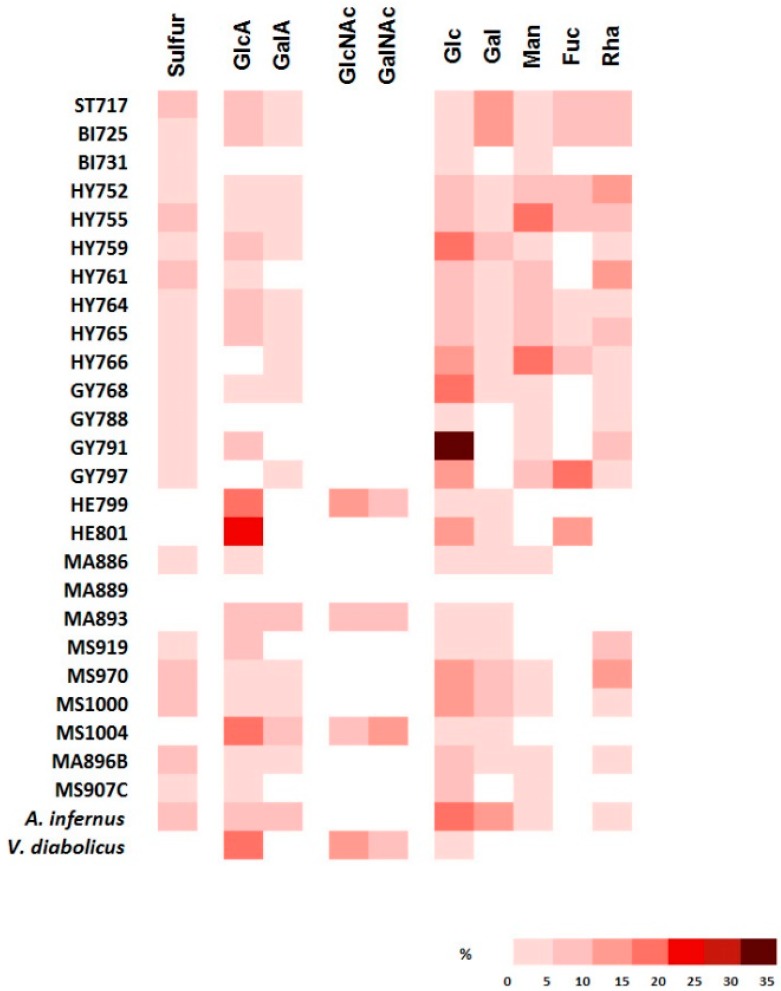
Sulfur content and monomeric composition (% *w*/*w*) of EPS for each strain. The EPS were extracted from a 100 mL culture broth.

The presence of both uronic acids and neutral sugars was assessed in all of the EPS by colorimetric assays (Table S2). Monosaccharide composition (GC analysis) revealed the presence of glucuronic and galacturonic acids (GlcA and GalA), *N*-acetylglucosamine, and *N*-acetylgalactosamine (GlcNAc and GalNAc) as well as neutral sugars such as glucose (Glc), galactose (Gal), mannose (Man), fucose (Fuc), and rhamnose (Rha) (Figure 2, Table S2). Among the EPS, four strains were particularly rich in uronic acids (GalA and GlcA) and *N*-acetylhexosamines (GlcNAc and GalNAc): HE800 EPS produced by *V. diabolicus*, and EPS produced by MS1004, MA893 and HE799. Nine other strains were also rich in uronic acids (>5% *w*/*w*); however, they did not contain any *N*-acetylhexosamines. Instead, they were rich in neutral sugars such as Glc and Gal that were present in GY785 EPS produced by *A. infernus* and EPS from MS1000, GY768, and GY791. In addition, some EPS exhibited important amounts of Fuc (ST717, BI725, and HE801) and Rha (ST717, BI725, HY765, GY791, MS919, and MS970). The presence of Fuc has been shown to be very important for some biological activities [49]. Although considered as a rare sugar together with Rha [49], they have both already been described in EPS from *Pseudoalteromonas* and *Polaribacter* strains isolated from Antarctic sea water, and sea ice and ocean sediments [50,51] as well as in *Vibrio parahaemolyticus* EPS [52] and that of *Enterobacter* A47 [53].

Sulfur was found in the EPS from 21 strains in a range from 2.6–5.5% (*w*/*w*) (Figure 2, Table S2). It should be noted here that the four EPS composed of uronic acids and *N*-acetylhexosamines did not bear any sulfate group. Two sulfated EPS did not contain any uronic acid or *N*-acetylhexosamine (BI731 and GY788) and could therefore resemble sulfated mannans already identified in seaweeds [54]. *Pseudoalteromonas* sp. strain SM20310 has already been shown to produce a non-sulfated mannan [12]. The protein content of the EPS varied from 1% (*w*/*w*) for HE799 to 30.7% (*w*/*w*) for BI731. Proteins were most likely co-extracted with the EPS from the supernatant and were thus considered as contaminants of the EPS preparation. Indeed, since the EPS had a negative charge due to uronic acid and sulfate groups, their ability to bind and trap several compounds was very high [1,13]. Glucose can also be co-extracted with the EPS when residual glucose is present in the culture broth, but has also been described as a component of several heteropolysaccharides [21,44].

The average molecular weight ranged from 1.4 for MS1004 to 9.2 × 10^6^ g·mol^−1^ for MS919. Thus, the EPS produced by the selected marine bacteria were high molecular weight (Table S2). The polydispersity index was close to 1, which emphasized that the EPS produced were highly monodisperse.

No typical monosaccharide was identified in the EPS produced by the MA889 strain, despite its high recovery yield of 0.55 g·L^−1^ and colorimetric assays showing uronic acid and neutral sugar presence (Figure 2, Table S2). It was thought that the cleavage by methanolysis might be incomplete, as already described for certain glycosidic bonds [55,56,57]. In addition, some monosaccharides have been shown to be degraded depending on methanolysis conditions [58].

### 3.3. Phylogenetic Analysis

New 16S rRNA sequences obtained in this study are reported in Supplementary File S4. The blast similarity search on the NCBI database for all 51 strains is reported in Table S1 where it was revealed that the initial strain list contained 28 *Alteromonas* sp. bacteria (15 were selected for in depth analysis), 12 *Pseudoalteromonas* sp. strains among which five were selected, and five strains of *Vibrio* sp. (all EPS were studied). In addition, the *Leclercia*, *Psychrobacter*, *Shewanella*, *Staphylococcus,* and *Tenacibaculum* species had only one representative in the collection. Among them, the only species having shown EPS production were *Psychrobacter* and *Staphylococcus,* but the EPS was too low and these strains were not studied further. This suggested that the main deep-sea bacteria producing EPS belonged to the gamma-proteobacteria *Vibrio*, *Alteromonas,* and *Pseudoalteromonas* species.

**Figure 3:**
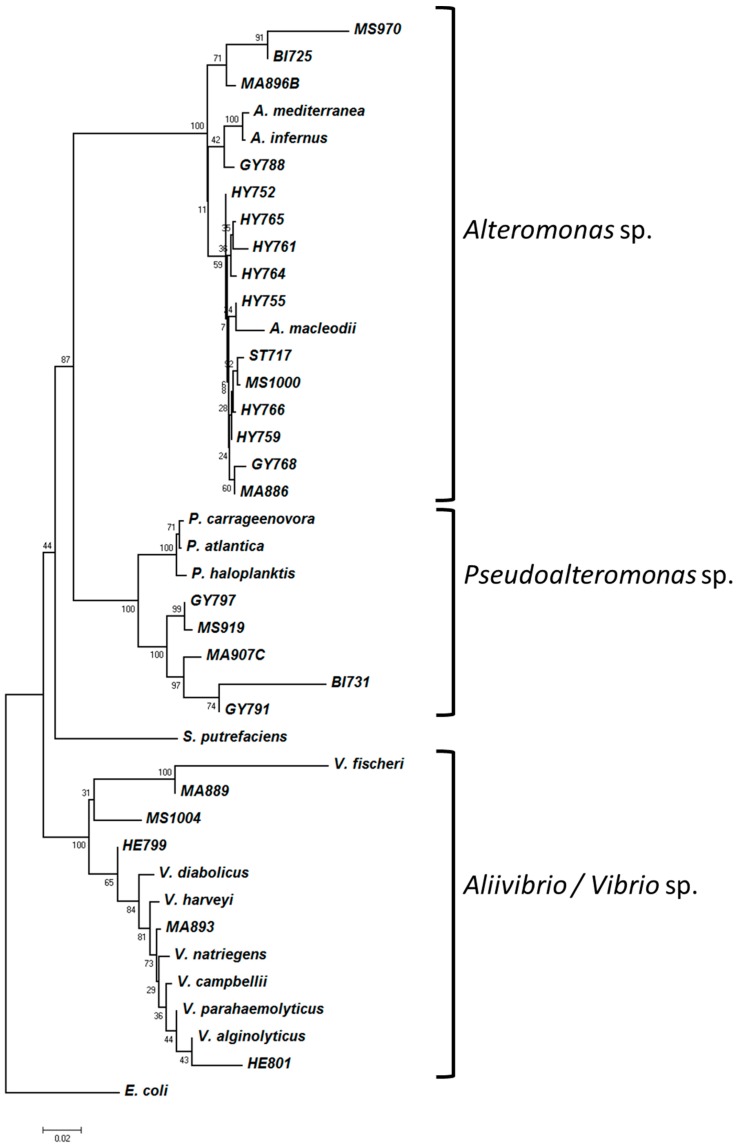
Phylogenetic relationship of the EPS-producing strains studied.

**Figure 4:**
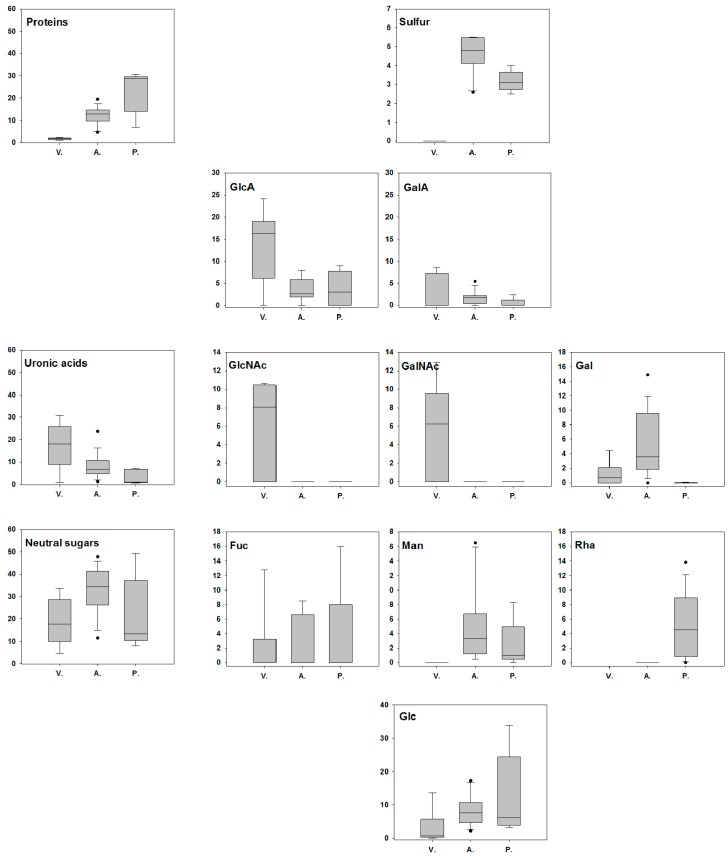
Box plot representation of the composition of EPS (% *w*/*w*) as a function of strain phylogeny: **V.** stands for *Vibrio* sp., **A.** for *Alteromonas* sp. and **P.** for *Pseudoalteromonas* sp. In the boxes, the 25th, 50th, and 75th percentiles are indicated by the bottom, middle and top lines, respectively. Whiskers show the 10th and 90th percentiles. Individual dots are the outliers. EPS were extracted from 100 mL culture broth and analyzed by colorimetric assays (sulfur, proteins, uronic acids, and neutral sugars) and GC (monomers).

To infer the phylogenetic relationships of EPS-producing strains, a tree was constructed using the 16S rRNA genes. 16S rRNA gene sequences of 15 reference strains were retrieved from the NCBI (Table S3) including *V. diabolicus* and *A. infernus*. A phylogenetic tree was built with these 40 16S rRNA sequences (Figure 3). The three main phylogenetic groups were indicated. The analysis of EPS composition of each taxonomic group revealed that none of the EPS produced by *Vibrio* sp. was sulfated (Figure 4). On the other hand, they contained higher amounts of uronic acids (GlcA and GalA) and, in contrast with *Alteromonas* sp. and *Pseudoalteromonas* sp. EPS, possessed *N*-acetylhexosamine monomers (GlcNAc and GalNAc). Glucose and protein amounts were higher in EPS from *Alteromonas* and *Pseudoalteromonas* strains, suggesting that if they were not components of the glycopolymer, they could be trapped by the sulfate negative charge (Figure 4). For each EPS, when present, the GlcA amount was higher than the GalA. Overall, the *Vibrio* EPS appeared to be poor in neutral sugars excluding *N*-acetylhexosamines. Fucose was detected in each taxonomic group, while mannose was absent from all *Vibrio* EPS, and rhamnose was mainly present in *Pseudoalteromonas* EPS (Figure 4). In conclusion, we observed that general traits of the EPS composition were specific to the bacterial genera.

Purified EPS from marine bacteria are usually heteropolysaccharides bearing functional groups such as hydroxyl, carboxyl (from uronic acid), and *N*-acetylamine groups. Some rare sugars such as fucose or rhamnose containing methyl groups may also be encountered [21]. In addition, inorganic (sulfate, phosphate) or organic (such as pyruvate, lactate, glycerol-1-phosphate (Gro1P)) acids are also frequently identified in EPS [11,20,21,59,60,61]. The chemical composition seems to be species-dependent, even strain-specific [48]. Growth conditions (carbon and nitrogen sources, pH, temperature) may also have a great influence on the structural features of the EPS [62]. Our findings were consistent with the previous description of bacterial EPS.

The EPS of several *Vibrio* strains were shown to contain hexosamines (or amide groups): *V. fischeri* [63], *V. harveyi* VB23 [64], *V. furnissii* VB0S3 [65], *V. parahaemolyticus* [52], and *V*. *neocaledonicus* [66]. Therefore, *N*-acetylhexosamines could be a common trait to *Vibrio* strains. Other EPS from *Pseudoalteromonas* bacterial strains have also been described [13] as well as from *Alteromonas* strains [18,20,21], which were all sulfated.

So far, EPS production has been mainly found in three main genera (*Vibrio*, *Alteromonas*, *Pseudoalteromonas*) and bioprospecting continues as marine environment contains a high microbial diversity [67]. In other studies, several EPS producing marine bacterial strains have also been identified in the genera *Halomonas* and *Enterobacter* [11,68], in *Pseudomonas* and *Paracoccus* strains isolated from French Polynesia microbial mats [61] as well as in *Cobetia marina* [69].

Previous data have suggested that the EPS may be strain specific [48]; however, we demonstrated that at the level of the bacterial genus, EPS structural features exhibited general specific traits. This further suggests that similar biosynthetic gene clusters might be responsible for the production of such carbohydrates. Little variation in gene regulation or expression, as well as in encoded proteins could result in slight molecular structural variability of the EPS. It has already been shown that the *syp* gene cluster encoding the biosynthesis of EPS in *V. fischeri* and *V. diabolicus* could be widespread among Vibrionaceae [70]. 

## 4. Conclusions

In this study, 51 new bacterial strains isolated from deep-sea hydrothermal vents were investigated for their ability to produce anionic polysaccharides. Chemical and phylogenetic analyses indicated that the majority of the EPS produced were anionic in nature and the producing strains in the collection mainly belonged to *Alteromonas*, *Pseudoalteromonas,* and *Vibrio* strains. Therefore, the marine environment appears to be a promising source to isolate new polysaccharides sharing common features with animal GAGs. Some EPS (MA886, MA889) may have unusual chemical structures and further studies are necessary to investigate them.

In the search for polysaccharides putatively mimetic of GAG, such as HA and heparin, HMW polyanionic carbohydrates are considered of high interest. Our data suggested that the best sources for HA-like EPS were bacterial members of the *Vibrio* species. On the other hand, sulfated carbohydrates of lower molecular weight can be alternatives to heparin-like GAG, and *Alteromonas* and *Pseudoalteromonas* appeared to be good candidates for such high-value carbohydrates. After the selection of bacterial strains of interest in the production of EPS of a particular composition, optimization of the production can be carried out. In addition, carbohydrate specific features can be further targeted by in vitro modification using a range of physical, chemical, mechanical, and even enzymatic methods.

## Supplementary Materials

The following are available online at www.mdpi.com/2076-2607/5/3/63/s1, Table S1: List of bacterial strains studied in this work. EPS results from the screening step are also indicated as well as the phylogenetic identification, Table S2: Chemical features of EPS. Colorimetric assays, GC results and molar mass. nd: not detected, Prot: protein, UA: uronic acids, D: polydispersity, EC: standard deviation, Yield is in g·L^−1^, all values are in weight percentage (% *w*/*w*), Table S3: 16S rRNA gene sequences of reference strains retrieved from NCBI, File S4: 16S rRNA genes sequenced in this study.
